# The contribution of simulated patients to meaningful student learning

**DOI:** 10.1007/s40037-021-00684-7

**Published:** 2021-10-12

**Authors:** Annelies Lovink, Marleen Groenier, Anneke van der Niet, Heleen Miedema, Jan-Joost Rethans

**Affiliations:** 1 Department of Technical Medicine, University of Twente, Twente, The Netherlands; 2 Department IQ Healthcare, Radboud University Medical Center Nijmegen, Nijmegen, The Netherlands; 3 Skillslab, Faculty of Health, Medicine and Life Sciences, Maastricht University, Maastricht, The Netherlands

**Keywords:** Simulated patients (SPs), Medical communication, Student learning, Meaningful learning

## Abstract

**Introduction:**

Communication training with simulated patients (SPs) is widely accepted as a valuable and effective means of teaching communication skills. However, it is unclear which elements within SP-student encounters make these learning experiences meaningful. This study focuses on the SP’s role during meaningful learning of the student by giving an in-depth understanding of the contribution of the SP from a student perspective.

**Methods:**

Fifteen bachelor Technical Medicine students were interviewed. Technical medicine students become technical physicians who optimize individual patient care through the use of personalized technology. Their perceptions of meaningful learning experiences during SP-student encounters were explored through in-depth, semi-structured interviews, and analyzed using thematic analysis.

**Results:**

Three main themes were identified that described what students considered to be important for meaningful learning experiences. First, SPs provide *implicit feedback-in-action.* Through this, students received an impression of their communication *during* the encounter. Implicit feedback-in-action was perceived as an authentic reaction of the SPs. Second, implicit feedback-in-action could lead to a process of *reflection-in-action*, meaning that students reflect on their own actions *during* the consultation. Third, interactions with SPs contributed to students’ *identity development*, enabling them to know themselves on a professional and personal level.

**Discussion:**

During SP encounters, students learn more than just communication skills; the interaction with SPs contributes to their professional and personal identity development. Primarily, the authentic response of an SP during the interaction provides students an understanding of how well they communicate. This raises issues whether standardizing SPs might limit opportunities for meaningful learning.

**Supplementary Information:**

The online version of this article (10.1007/s40037-021-00684-7) contains supplementary material, which is available to authorized users.

## Introduction

Health professionals’ strong communication skills have been linked to effective patient interviewing, enhanced patient and professional satisfaction and improved patient outcomes [1–3]. Simulated patients (SPs) play an instrumental role in teaching communication skills. SPs are lay people or actors trained to portray a patient with a specific condition in a realistic way [4, 5]. Most medical schools include SP programs in their curriculum to create an effective learning environment[6].

Teaching communication skills with SPs in a simulated setting can optimize students’ approaches toward meaningful learning. During communication training, the student’s interaction with the SP facilitates the learning experience. SPs provide specific advantages over working with peers or real patients. For example, there are opportunities to experiment with different approaches, to standardize patient cases across student encounters, and to customize students’ learning needs [4–7]. However, what exactly makes the interaction with SPs a meaningful learning experience to students remains unclear.

There are multiple perspectives on meaningful learning [10, 11]. According to the Experiential Learning Theory, experience is the core element in a learning process [8, 9]. This experience can stimulate more or less meaningful learning for students. Ausubel described meaningful learning as learning that is well anchored and integrated in the cognitive structure of learners, in contrast to rote learning such as reproduction-oriented learning [11]. Meaning-oriented learning can be seen as deep learning that stimulates reflection [10]. In educational programs, this reflection is often facilitated retrospectively to support meaningful learning. However, according to the Performance-Relevant Information (PRI) theory, there can already be information that is deemed relevant to the learner in the interaction itself, so *during* an event [12]. PRI theory focuses on how learners interpret their performance in terms of what is relevant for their learning. It includes all types of information, from explicit feedback to the more implicit interpretations [12]. The study by Van der Leeuw et al. about workplace-based learning in clinical context described an implicit form of feedback such as interruptions and questions that served as a potential motivator for changing future behavior [12]. Also, SP responses during SP interactions can provide students with information about their performance during the interaction, which can facilitate deep learning by stimulating reflection. We propose that this reflection likely occurs not only after, but during the activity. Schön called this *reflection-in-action*, meaning that we can think about what we are doing, while we are doing it [13]. In line with Ausubel and Schön, we defined meaningful learning as deep learning that is anchored and integrated in the cognitive structures of a student and is stimulated by reflection [11, 13].

Given that the SP-student interaction provides a learning experience, we propose that this experience might stimulate meaningful learning. For example, different SPs can have different reactions during a medical interview where the student tries to empathize with the patient, which could lead to different learning experiences. If a student were to say: “I understand how you feel about it” in one instance the SP (1) might respond incredulously “Do you?!” whereas a second SP (2) might say “Yes, I am really sad about it”. These reactions can result in two different subsequent interactions and learning experiences for a student. From SP1, the student might learn to avoid responding to emotions in this way, while with SP2 the student might be encouraged to explore the emotional response of the SP in-depth. However, whether both situations lead to* meaningful* learning experience from a student perspective remains unclear. Therefore, our research question is: How does the SP contribute to meaningful learning of the student *during* a specific SP-student encounter, from a student perspective? By better understanding the contribution of SPs during the SP-student interaction, SP-mediated learning can be enhanced. Based on meaningful learning [11], Experiential Learning Theory [8, 9] and Performance-Relevant Information [12, 13], we expect SP-student encounters to potentially facilitate meaningful learning experiences by simultaneously providing relevant information for the learner during the encounter.

## Method

### Setting

This study was conducted at the undergraduate Technical Medicine program of the University of Twente (the Netherlands) and ethically approved by the Netherlands Association of Medical Education [NMVO, NERB number 1050]. A technical physician is a healthcare professional who optimizes individual patient care through use of personalized technology [14]. As technical physicians are legally licensed to perform medical interventions, teaching medical communication skills plays an essential role in the three-year bachelor program. SPs are engaged to train and assess these communication skills. SPs at the University of Twente are lay people who have been trained to portray a patient in a realistic way. The communication program involved 15 SP-student encounters per student throughout the bachelor curriculum, in which students practiced basic to advanced communication skills, such as breaking bad news. Students received feedback on their performances from peers, teachers and SPs.

### Design

We selected a qualitative approach and conducted semi-structured individual interviews with 15 bachelor technical medicine students. We explored their perceptions of meaningful learning experiences during SP-student encounters. We used thematic analysis to analyze the data [15].

### Participants

We used purposive sampling to identify participants. Eligible participants included students who had completed the 3‑year communication program of technical medicine in 2018. Although this meant that interviews could not take place directly after SP consultations, all students had multiple experiences with SPs and were better able to look back on the whole SP program. All students [*n* = 90] who completed the communication program were verbally invited to participate by AL at the start of a lecture, followed by an email invitation. We interviewed 15 student volunteers, nine men and six women, aged between 20–23 years.

### Interviews

We developed a semi-structured interview guide informed by the key research question, which queried participants’ meaningful learning experiences and learning moments to gain insight into their meaningful learning during SP interactions. According to PRI theory, the emphasis during the interviews was on information that was deemed relevant to the student during the SP interaction [12]. Based on a pilot interview the interview schedule was refined (see Appendix 1 of the Electronic Supplementary Material [ESM]). One researcher (AL), a lecturer at the University, conducted all 15 interviews in Dutch, which lasted 40 to 60 min. AL’s role as lecturer was considered during recruitment such that only students that she did not assess were selected for participation. All interviews were audio recorded and transcribed verbatim.

### Analysis

We utilized thematic analysis [15]. Three researchers (AL, MG, AvdN) independently coded the first three interviews applying general principles of open coding, using the Atlas.ti. software program. Themes were identified in individual interviews by repeated reading and constant comparison of the individual interviews with the research question. Data were reviewed jointly followed by collaborative discussion among all researchers about the appropriateness of the themes. Meetings to compare and refine the analysis occurred after coding every three to four interviews. Codes and categories were discussed and clarified before the interviews were re-read and re-analyzed using the final coding guide. Saturation was reached after 13 of 15 interviews.

All authors adopted a reflexive attitude to discuss their initial observations of the data in relation to their own interests and biases. All researchers discussed reflexivity to identify their potential biases and presuppositions. They considered their own occupational roles and how these might affect their initial reading of the data. In the spirit of reflexivity, we provide the authors’ relevant backgrounds. Researcher and interviewer AL is a lecturer and SP educator at the Department of Technical Medicine with 12 years of experience working with SPs. MG is a lecturer and researcher at the Department of Technical Medicine with substantial experience in human and non-human simulation education. HM is the director of education for technical medicine. She designed the technical medicine educational program. AvdN has extensive research experience using qualitative methodologies. JR is a professor in the field of human simulation and has worked with SPs since 1985.

## Results

We identified three main themes derived from 17 code groups. Meaningful learning experiences reported by the students were characterized by *implicit feedback-in-action* and *reflection-in-action*, at times contributing to *identity development*. Implicit feedback-in-action and reflection-in-action are closely related processes during the SP-student encounter, as they take place in the flow of the interaction. In the following sections, we describe the three themes separately and provide context by presenting quotes from a variety of student participants as indicated by S#.

### Implicit feedback-in-action as authentic reaction

Students indicated that SPs contribute to their meaningful learning experiences by receiving implicit feedback-in-action from the SP during the encounter. Through this implicit feedback-in-action, students get an impression of how well they communicated during the encounter. Implicit feedback-in-action is described, in this study, as the reaction of the SP during the interaction integrated in the role play. For example, students pointed out moments when the SP, in her/his role as patient, let the student know that the conversation was not going well. This was not through feedback the SP provided afterwards, but during the conversation while the SP was fully engaged in the role play. Students described the reaction of the SP to their interventions during the encounter as a form of feedback. A student argued: “*Yeah, and everything you (the student) say, they (the SP) react to. And that is actually a direct form of feedback on your intervention”* (S3).

This form of implicit feedback is not only verbal but also non-verbal. In about half of the cases where implicit feedback-in-action was described, students perceived this implicit feedback-in-action as corrective. Through the SP’s reaction, they learned that the conversation was not going well.

Students also described a positive form of implicit feedback-in-action, which was perceived as affirmative. Through the SP’s reactions, they could intuit that they were performing well:

*But also simulated patients who are very closed-off at the beginning and after a while they begin to open up. Or they make more eye contact, that kind of thing. […] I think there was one patient last year who just kept looking at her phone; however, at a certain point I noticed that was getting less. *(S1)

Students perceived this implicit feedback-in-action by the SP as an authentic and realistic reaction to their actions and attitude, incorporated in the role play: “*It is real to me. They (the SPs) take the role play quite seriously. In my opinion, it involves real emotions. At least they express the emotions as if these are real emotions*” (S1).

### Reflection-in-action

The different expressions of implicit feedback-in-action can facilitate a process of reflection-in-action for the student. Students indicated that the implicit feedback-in-action of the SP during the encounter provoked a process of immediate self-reflection:

*If a simulated patient, in their role as a patient, makes clear to me that I’m not doing the right thing (implicit feedback-in-action), I appreciate that. It gives me a bit of a shock, and I think: oh yes, that’s true. And then you realize that is how a real patient might react (reflection-in-action). So that is really beneficial for my learning.* (S11)

Additionally, students described implicit feedback-in-action and reflection-in-action as closely related, as is clear in the following example where the implicit feedback-in-action by the SP caused a process of reflection-in-action by the student:

*For example, during a consultation with the patient and their partner. At a certain point you can become completely focused on the partner. And when the simulated patient points out that he is the patient (implicit feedback-in-action), you become aware of the situation. Then you think “Of course, that’s true, I am only talking to the partner now”. In that sort of situation, you subconsciously know that you are only talking to the partner and not to the patient, but because the patient says it explicitly, then you become really aware of it! (reflection-in-action)* (S12)

Students described that they not only reflected on their own interventions, but that they also adapted their behavior during the same encounter. They mentioned that they could immediately apply the SP’s implicit feedback, after a short moment of reflection-in-action during the consultation:

*Yes, especially the tiny facial expressions of the simulated patient (implicit feedback-in-action). They make you think, I’m saying something wrong (reflection-in-action). Or you say something and you notice that it is not right. Then you have to change gears and modify your reaction straight away.* (S10)

### Identity development

On one hand, implicit feedback-in-action and reflection-in-action were linked to concrete situations during the SP-student encounter. On the other hand, there appears to also be an overall level of meaningful learning that students described. Students indicated that multiple SP interaction contributed to their identity development. Identity development was mentioned on two closely related yet slightly different levels, a professional and a personal level. Students described that during the experience with an SP, they learned how they reacted and behaved in a particular situation, and the experience provided them with a sense of control over their own behavior as a professional: “*I think it is quite good that I know how I react in certain situations. […] So that it will not surprise me in my first real consultation, that I will be nervous. I now know, I can handle it*” (S9). Furthermore, students mentioned that over the years, after SP multiple experiences, they learned how to behave in a professional way: “*Over the years, through contact with all those patients, I have learned that is it is better to think before speaking*” (S6). Additionally, students indicated that they developed their identity on a personal level, meaning that they got to know themselves better as a person through SP encounters: “*I thought it was fun and also instructive. Not only for me as a technical physician, but also for me personally, for who I am as a person*” (S12).

## Discussion

**Fig. 1:**
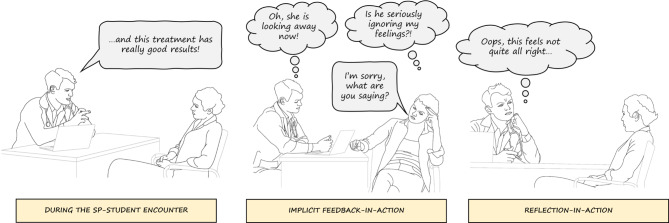
Interaction between student and simulated patient

In this study, we examined the contribution of the SP to students’ meaningful learning during SP-student encounters, from their own perspective. We were interested in what aspects *during* the SP-student interaction make the learning experience meaningful to a student. Meaningful learning experiences reported by the students were characterized by implicit feedback-in-action and reflection-in-action, at times contributing to identity development. Implicit feedback-in-action and reflection-in-action in this study are described as two different, but closely related processes during the SP-student encounter, as becomes clear in Fig. 1 about SP-student interaction.

SPs’ reactions provide students with feedback on their behavior and attitude. This gives students the opportunity to reflect on their actions and can be seen as a feedback loop during the SP-student encounter, facilitated by SPs. Eppich et al. [16] described a similar process during the interaction between supervisors and doctors-in-training, where the trainee perceived feedback that was not explicit but ‘disguised’ to be an implicit form of feedback serving as a potential motivator to changing future behavior. As described in the introduction, this is called PRI [12]. Our results show that students described implicit feedback-in-action, a form of PRI, provided by the SPs as highly relevant for the learning and reflection process. This implicit feedback-in-action triggers a process of reflection-in-action: students are thinking about what they are doing while they are doing it [13]. Students indicated that they were sometimes even able to change their behavior right away or gained confidence that they were on the right track. In communication education, great emphasis is placed on feedback after a SP-student encounter. Our findings have potential to encourage teachers and students to also focus on the meaningful learning of the student on the spot, during the flow of the interaction. Thus, providing students the opportunity to learn during an encounter in which SPs provide feedback-in-action will be valuable. We therefore need to identify approaches to support SPs in giving feedback-in-action.

Meaningful learning experiences are not only important for refining students’ communication skills, but also contribute to students’ identity development. Students learn not only to communicate with patients, but also who they are as healthcare professionals. This also aligns with McLean et al.’s study in which SPs were interviewed about the students’ professional identity development. They found that SPs contributed to students’ professional identities by creating a supportive environment for honing skills, which they did through realistic role playing, making their bodies available, and by providing feedback as a patient [17]. Additionally, participants indicated that their identity development occurred on both a professional and personal level. Feedback during the event, therefore, has the potential for greater personal growth and learning [18].

A central characteristic of simulation-based (technical) medical education is its unique approach to making and learning from mistakes [19]. We noticed that moments of corrective feedback were fresher in students’ memories when asked about meaningful learning experiences. This suggests that, when looking back at the example in the introduction to this article, the reaction of SP1 has more potential to be a meaningful learning experience for the student, as corrective feedback is more likely to grab the student’s attention, leading to a moment of reflection-in-action. It might confront the student more in comparison with the reaction of SP2, which is affirmative. However, the interviews also revealed that it is not just a single sentence that makes an experience contribute to meaningful learning. It is the flow of the interaction that can facilitate a meaningful learning experience. In addition, it might be important to create a balance between emotional load associated with the experience and the professional lessons that can be learned [19].

Participants frequently mentioned SP authenticity. Timing and real emotions seem to play a role in the degree to which a student experienced the SP’s authenticity. It is not only the authenticity of the role play, but also the personal authenticity of the SP: the authentic, non-scripted, reaction of the SP that is integrated in the role play during implicit feedback-in-action. It is the SP, as an authentic person with an authentic response but still in the role play, who provides the student with feedback. Students described that they felt the SP’s real personal emotion, which evoked real emotions in them. As authenticity plays such an important role in SP-student interaction and meaningful learning experiences, it is important to recruit SPs able to maintain their authenticity during role play. Additionally, SPs should be stimulated to be authentic during role play while at the same time maintaining the right degree of standardization, even if this seems paradoxical. There might be a fragile balance between standardized role play and authentic responses during role play, which can create the learning experience for a student. In communication education great emphasis is placed on SP training and selection to achieve high standards of standardization. However, by doing so we may be losing the important aspect of authenticity, whereas authentic responses of the SPs can be of great impact for the learning experiences of the student. It is possible that with ‘overtraining’ and standardization of our SPs, we unknowingly and with the best intentions withhold meaningful learning experiences from our students.

## Limitations

This study is based on a single data source and a sample from one technical medical school. However, by describing the findings and the context in detail, explaining our sampling strategy and discussing our findings with existing literature from different settings, we promote transferability. Credibility could be enhanced by exploring this topic from different perspectives, including the perspectives of SPs. However, we used investigator triangulation and theory triangulation to promote trustworthiness. Furthermore, the interviews took place a few months after students had their last SP-student encounter. Interviewees therefore had to report from memory, which might have resulted in slightly different reports on their experiences compared with shortly after their last SP-student encounter. However, students now had more experience with multiple SPs, were better able to look back on the whole SP program and their response depended less on single experiences. In addition, the fact that students remembered learning moments after a time had passed could be an indication of meaningful learning anchored in their cognitive structures. Lastly, we did not observe the actual behavior during SP-student interactions. It would be worthwhile to observe the behavior during SP-student interaction and to analyze the thoughts and behaviors of the SPs and students with stimulated recall directly after an SP-student encounter.

## Conclusion

During SP-student encounters students learn more than just communication skills; the interaction with SPs contributes to their professional and personal identity development. It is the implicit feedback-in-action as an authentic response of the SP during the interaction that gives students an understanding of how well they communicate. By balancing authenticity and standardization in SP role play, students learn most from SP encounters. This study raises issues about the impact of too much standardization on this balance, which should addressed in SP training and selection.

## Supplementary Information

Appendix 1 contains the interview schedule for students about SP-student interaction
