# Supplementary material for: The contribution of simulated patients to meaningful student learning
Source: Perspect Med Educ. 2021 Oct 12;10(6):341–6. doi: 10.1007/s40037-021-00684-7 (PMC8633349; doi:10.1007/s40037-021-00684-7)
Supplement: Supplementary file 1 — Appendix 1 contains the interview schedule for students about SP-student interaction [file 40037_2021_684_MOESM1_ESM.docx]

Appendix 1

**Interview schedule for students about SP-student interaction**

**Semi-structured interview schedule**

**1. Questions about SPs in general**

1.1 You are now in your third year of your bachelor and you have a lot of experience with
education using simulated patients. How do you perceive the program with SPs so far?

1.2. What do you think of SPs within the VCPG* program?

1.3. How do you perceive the interaction with the SPs outside the consultation?

1.4. How do you perceive the interaction with the SPs during the consultation?

1.5. What do you think the role of the SP is in your learning process?
How do you see the SP? Do you see the SP as a patient or perhaps as a teacher?

1.6. Does age difference play a role in the interaction between you and the SP?
If so, how do you deal with this?

1.7. Do other differences play a role in the interaction between you and the SP, for example cultural differences, gender, power differences or (life) experience?
If so, how do you deal with this?

*VCPG = communication training

**2. Questions about the contribution of the SPs**

2.1 What did you learn from the consultations with SPs?
To what extent does the SP contribute to this?
How do you think the SP contributes to this?
How important is that for you?
Do you have any examples?


2.2. To what extent does a consultation with an SP differ from other educational situations?

2.3. What are the important learning moments during the consultations with an SP? Can you describe them?

2.4. What do you think that the SP contributes to those moments?

2.5. Do you ever feel that an SP consciously creates a learning moment for you?
Do you have any thoughts about that?
Do you have any examples?

2.6. Did you ever have the idea that the SP lost his/her way and that created a moment that was actually negative for your learning? In other words, were there negative learning experiences, and what caused them?

**General questions**
Participant number:
Age:
Gender:
Academic year:
Date:
